# Skinfold Thickness Measurement in Term Nigerian Neonates: Establishing Reference Values

**DOI:** 10.1155/2018/3624548

**Published:** 2018-01-01

**Authors:** Olanike Abosede Olutekunbi, Adaobi Uzoamaka Solarin, Idowu Odunayo Senbanjo, Elizabeth Aruma Disu, Olisamedua Fidelis Njokanma

**Affiliations:** ^1^Paediatrics Department, Gbagada General Hospital, Gbagada, Nigeria; ^2^Lagos State University Teaching Hospital, Ikeja, Nigeria

## Abstract

Skin fold thickness (SFT) measurement is a reliable, cheap, simple, noninvasive method of body fat estimation at all ages including the neonatal period.* Objective*. To determine reference values of biceps, triceps, subscapular, and suprailiac skinfold thickness measurements in term Nigerian newborns.* Method*. A prospective cross-sectional study over a six-month period (Dec 2010–May 2011) was carried out on term and healthy neonates delivered between 37 and 41 weeks. The anthropometric measurements were taken within the first 48 hours of life including the skinfold thickness. The skinfold thickness measurements were taken at four sites, namely, triceps, biceps, subscapular, and suprailiac, using Harpenden skinfold calipers. The mean of two readings was recorded.* Result*. A total of one thousand one hundred and sixty-eight neonates were studied. The birth weight ranged between 2000 g and 5000 g with a mean birth weight of the neonates at 3259 ± 470 g. The mean birth weight of the males (3339 ± 0.45) was significantly higher than that of females (3200 ± 0.44) (*p* < 0.0001). Female neonates had higher mean values of triceps, subscapular, and suprailiac skinfold thickness (*p* < 0.001, resp.) while male neonates had higher mean value of biceps skinfold thickness (*p* = 0.008). Females also had higher mean values of the sum of skinfold thicknesses at all four sites and the sum at the two truncal sites at every stratified gestational age.* Conclusions*. The sex specific percentile chart developed for skinfold thickness measurements can be used to detect deviation from the reference population such that infants who are at risk of nutritional or health problems are identified early, and intervention is instituted promptly.

## 1. Introduction

Skinfold thickness (SFT) measurement is a reliable, cheap, simple, noninvasive method of body fat estimation at all ages including the newborn period [1]. It measures the thickness of subcutaneous fat at various sites of the body from which total body fat and hence contribution of fat to body mass can be estimated [1]. The relevance of its application is emphasized by the fact that nutritional status has a considerable impact on early and late neonatal outcome [1].

Data on skinfold thickness can be utilized in a number of ways. Firstly, they can be directly compared with reference values in an attempt to determine adequacy, deficiency, or excess. In doing this, results from one site or the summation of results from two or more sites may be compared with reference values [2]. In other words, interpretation of skinfold thickness values depends on availability of standard or reference charts. For example, standard values have been developed for Caucasian children and neonates [3, 4]. However, similar standards have not been developed for indigenous African neonates. The extent to which standards derived from Caucasian children are applicable to other ethnic populations is not established especially as racial factors are known to affect body size and proportions. As previously documented, anthropometric differences exist between newborns and people of different races [5]. Therefore, a local or regional study aimed at developing reference values for our newborn is of utmost importance because, intrauterine life and infancy are critical periods for growth development [4].

Skinfold thickness values can also be introduced into predictive mathematical formulae to derive body fat indices like percentage body fat and hence derive fat mass and fat-free mass [6]. Measurements from various sites can also be used in a prediction equation to estimate body density before conversion to percent body fat [7]. The application of SFT in neonates is based on the fact that there is good correlation of birth weight with total body fat mass in newborns. SFT has shown good correlation with total body fat mass in newborn, and body weight is the best independent predictor of body composition in preterm and term infants accounting for 84% of the variation in fat mass [1]. While the use of skinfold thickness measurement as a reliable method of body fat estimation in the newborn has been known for several years [8], it has not been sufficiently explored in Nigerian or African neonates. There is, thus, a dearth of information on its use in these newborns. This study was undertaken to contribute to knowledge of SFT in Nigerian neonates and further establish a reference value for the Nigerian neonate. Body fat composition in the newborn is of immense clinical importance as an index of nutritional status and can help identify malnutrition.

Skinfold thickness is measured in millimeters using special calipers [9, 10]. There are different types of calipers designed to exert a contact surface pressure of 10 g/mm^2^ [10]. The common ones are Holtain, Harpenden, Lange, and McGraw [11]. Sites of skinfold thickness measurement are sometimes classified into limb sites and central sites. Limb sites include biceps, triceps, and quadriceps and calf regions while central sites include pectoral, subscapular, abdomen, and suprailiac regions. One side of the body either right or left is usually used for consistency of results [12]. In children, four sites are commonly used, namely, triceps, biceps, subscapular, and suprailiac. However, triceps and subscapular skinfold measurement are recommended as the minimum number of skinfold measurements in pediatric practice [13–15].

Like every anthropometric measurement, error of measurement is a problem. The skill of the observer and the quality of caliper are key issues. To eliminate error, the author was trained to use the caliper by an anthropometrist, a certified member of the International Society for the Advancement of Kinanthropometry (ISAK). All measurement was taken by only one observer, the author.

*Why Skinfold Thickness Is Measured?* Birth weight has traditionally been used as the single most important factor for determining neonatal survival perhaps because of its ease of measurement. However, it does not provide all the answers as it may still fall within normal limits even when a fetus has suffered some degree of acute weight loss in pregnancy. Some healthy babies may be constitutionally small but not undernourished. It is thus important to look beyond birth weight alone in determining neonatal outcome. SFT measurements are more complicated but more realistic and body fat values obtained by SFT measurement have been validated with gold standards like dual energy X-ray absorptiometry (DEXA) [4]. 

*Aim of Study*. The aim of the study was to describe the pattern of and determine the reference values of skinfold thickness in term newborns at the Lagos State University Teaching Hospital, Ikeja, Nigeria.

## 2. Subjects and Methods

### 2.1. Study Location

The study was carried out in the lying-in wards of the Obstetrics and Gynecology Unit of Lagos State University Teaching Hospital which was temporarily moved to the newly built Maternal and Child Centers at Ifako-Ijaiye and Isolo General Hospitals both in Lagos State during the period of study. The predominant ethnic group in Lagos is Yoruba though other Nigerian ethnic groups are well represented in the state.

Neonates were consecutively recruited from the lying-in wards (as they room with their mothers) until the desired sample size was attained from the 1 December 2010 to the 31 May 2011. Neonates delivered at full term (37 weeks to 41 weeks), singleton babies, and babies apparently well within the first 48 hours of life were included in the study while, babies with gross congenital defect, multiple births, and babies whose parents do not give their consent were excluded from the study.

Infants of diabetic mothers were not excluded from this study as we set out to investigate the relationship between birth weight and skinfold thickness in term newborns. Also, the incidence rate of diabetic pregnancies is reportedly low at about 0.64/1000 births/year [16]. The use of a chart based on a population with low incidence or low prevalence of risk factors can be safely acceptable [17].

An informed written consent was obtained from each mother. The study protocol was approved by the Lagos State University Teaching Hospital Ethical Review Committee.

### 2.2. Research Instruments

The instruments used include Harpenden® skinfold caliper, and a self-designed pro forma used to collect relevant information, an electronic weighing scale to measure birth weight, and an infantometer to measure the length of the neonate. The pro forma was validated by the certified anthropometrist a certified member of the International Society for the Advancement of Kinanthropometry (ISAK) who trained one of the researchers for six months and ensured proficiency before the actual study was conducted.

### 2.3. Methods

The neonate was examined thoroughly within the first 48 hours of life by the researcher for evidence of congenital malformation and signs of illnesses like pyrexia, jaundice, hepatomegaly, and cardiac murmur.

Birth weight was measured with the baby being nude using an electronic weighing scale (KINLee), which is accurate up to 2000 grams in 5-gram increments. The weighing scale was regularly calibrated after every 50th use according to the manufacturer's manual.

The gestational age of the baby was calculated from the date of the mother's last normal menstrual period. Where this was not known, the report of an early ultrasound scan done in the first trimester of pregnancy was used to estimate gestational age [18]. Modified Ballard's scoring was done where the last normal menstrual period and early obstetric ultrasound scan report were not available.

The length of the neonate was taken using an infantometer which is an instrument with a firm and flat or horizontal calibrated surface with two ends, a fixed end and a movable end. The infant is laid supine on the infantometer with the head placed at the fixed end of the infantometer in such a way that the occiput and the pupils are on the same vertical plane with the feet placed on the movable end and lower limbs extended gently but firmly held down by an assistant. The length (which is the distance between the two ends) is read off the calibrated horizontal surface in centimeters and to the nearest 0.1 centimeters [19]. The mean of two readings was taken and recorded.

The skinfold thickness method is based on measuring a pinch of skin precisely at several standardized sites on the body to determine the subcutaneous fat layer.

For the biceps and triceps skinfolds, the landmark was determined by measuring the mid distance between the acromion process of the right humerus (shoulder) and the olecranon process (elbow) of the same limb. The mid distance was marked on the skin anteriorly to measure the biceps skinfold and posteriorly to measure the triceps skinfold with the arm by the side of the body.

Subscapular skinfold thickness (SBS) is measured 1-2 cm below the inferior angle of the scapular. Skinfolds were raised over this point and held throughout the measurement with the caliper applied at right angle to the raised fold and the reading on the dial taken allowing full pressure of the caliper by a complete release of the trigger. The reading was made approximately 2-3 seconds after application as it is known that the fold cannot be held too long as the subcutaneous fat may be compressed. This was done with the neonate sleeping or resting on the mother's chest.

Suprailiac skinfold (SPS) is raised immediately superior to the iliac crest in line with the natural angle of the iliac crest at the anterior axillary line [20]. The caliper was applied at right angle to the raised skinfold for measurement with the neonate lying down on their side.

Measurement was made by lifting the skin with the thumb and index finger with care being taken to exclude any underlying muscle [4]. This was done with the baby sleeping calmly on the bed, the right arm by the side, or carried by mother on her chest with the right arm hanging by the side. A double fold of skin was raised over landmarks on the skin by a pinching, slight rolling action of the left thumb and index finger of the researcher. The fold was raised perpendicularly to the surface of the body at the measurement site and the amount of skin raised formed a fold with parallel sides. The fold was grasped firmly and held throughout the measurement. The caliper was applied at right angle to the raised fold at all times and the reading on the dial was taken after allowing full pressure of the caliper by a complete release of the trigger. The reading was taken approximately 2-3 seconds after application.

All the skinfold measurements were taken on the right side of the body for consistency [12, 21], when skin was dry and lotion-free and within the first forty-eight hours of life. The mean of two measurements was taken for all the anthropometric measurement.

The following indices were calculated from the skinfold measurement.

- Sum of skinfold measurements ∑SFT = (TS + BS + SBS + SPS).
- Central to total skinfold measurement ratio: SPS + SBS/∑SFT.

Quality assurance was ensured by ensuring that all skinfold thickness measurements were taken by one person who had been trained by a certified member of the International Society for the Advancement of Kinanthropometry (ISAK). Two measurements were taken at each site at least 15 seconds apart and the mean of the two readings was recorded. Calipers are cleaned before and after use on subjects and dial indicator at zero before measurement is taken.

### 2.4. Sample Size Calculation

Sample size was calculated using the formula [22]

(1) $$\begin{array}{c} n=\frac{{\left(z\times \sigma \right)}^{2}}{{\left(E\right)}^{2}}, \end{array}$$

where we have the following.

 
*n* is desired sample size.
 
*z* is standard normal deviation usually set at 1.96 and corresponds to 95% confidence level.
 
**σ** is standard deviation of the population sample as reported in an earlier study [1].
 
*E* is mean (of the sum of skinfold measurements from an earlier study) [1] × 0.05 because the degree of accuracy is set at 0.05.

Gestational age was stratified and the formula was used to calculate the number of neonates recruited for the study at each gestational age and for each gender.

**Table:** 

Boys	Girls
37 weeks, $n=\frac{{\left(1.96\times 3.38\right)}^{2}\;}{{\left(14.45\times 0.05\right)}^{2}}=84$	37 weeks, $n=\frac{{\left(1.96\times 2.89\right)}^{2}\;}{{\left(13.99\times 0.05\right)}^{2}}=65$
38 weeks, $n=\frac{{\left(1.96\times 2.86\right)}^{2}}{{\left(14.33\times 0.05\right)}^{2}}=61$	38 weeks, $n=\frac{{\left(1.96\times 3.07\right)}^{2}}{{\left(15.18\times 0.05\right)}^{2}}=63$
39 weeks, $n=\frac{{\left(1.96\times 2.89\right)}^{2}}{{\left(15.08\times 0.05\right)}^{2}}=56$	39 weeks, $n=\frac{{\left(1.96\times 2.77\right)}^{2}}{{\left(15.37\times 0.05\right)}^{2}}=49$
40 weeks, $n=\frac{{\left(1.96\times 2.77\right)}^{2}}{{\left(15.17\times 0.05\right)}^{2}}=51$	40 weeks, $n=\frac{{\left(1.96\times 2.91\right)}^{2}}{{\left(15.58\times 0.05\right)}^{2}}=54$
41 weeks, $n=\frac{{\left(1.96\times 2.74\right)}^{2}}{{\left(15.33\times 0.05\right)}^{2}}=49$	41 weeks, $n=\frac{{\left(1.96\times 2.99\right)}^{2}}{\;{\left(16.18\times 0.05\right)}^{2}}=52$
Total = 301	Total = 283
Sum total = 584 (five hundred and eighty-four).	

 The sample size was doubled to total of 1168 (one thousand one hundred and sixty-eight) subjects because the larger the sample size, the more the probability of its being representative of the population from which subjects were selected.

### 2.5. Ethical Considerations

The study was approved by the Lagos State University Teaching Hospital Ethical Review Committee (LREC/10/08/144).

### 2.6. Data Analysis

The collected data was stored for analysis in an SPSS version 17.0 file. Statistical measures like means, standard deviations, ranges, and ratios were calculated using this software. Chi square analysis, Pearson's correlation coefficient, and Student's *t*-test were used where appropriate for comparing discrete and continuous data, where applicable. Multiple regression analysis was carried out to determine predictors of skinfold thickness. Probability (*p* value) less than 0.05 was interpreted as statistically significant (Table 8).

## 3. Results

Table 1 shows the mean birth weights of the neonates according to their sex and gestational ages. The mean weight increases with increasing gestational age in both sexes and the overall mean was significantly higher in males compared to females (*p* < 0.001).

Table 2 shows the distribution of various skinfold measurements in the subjects according to gestational age and sex. The highest mean SFT was observed at the subscapular site followed by triceps, biceps, and suprailiac sites in that order. The pattern was the same in both males and females.

The overall mean triceps SFT was higher in females at each gestational age, being most significant at 39 weeks (*p* = 0.021) and 40 weeks (<0.001). A similar pattern was noted with respect to subscapular SFT with an additional observation of significant difference at 37 weeks. Further, at all gestational ages, the mean suprailiac SFT was significantly higher in females. A different trend was however noted with respect to biceps SFT in which males had higher values at all gestational ages except at 40 weeks. The overall figure for males was significantly higher (*p* = 0.008) and this was reflected at 38 weeks (*p* = 0.029) and 39 weeks (*p* = 0.035).

The SFT at the subscapular and suprailiac sites were summed up in Table 3 to give an index of central fat (SBS + SPS). Observed values were consistently higher in females at each gestational age with significant differences being observed at 39 weeks (*p* < 0.001), 40 weeks (*p* < 0.001), and 41 weeks (*p* = 0.002) and overall (*p* = 0.003).

The pattern of overall central to total skinfold thickness ratio, that is, the sum of the subscapular and suprailiac skinfold thicknesses divided by the total sum of SFT at the four sites (SBS + SPS)/∑SFT as shown in Table 4 was similar to that of the central fat. The central fat ratio did not show any particular pattern of variation with gestational age.

**Figure 1:**
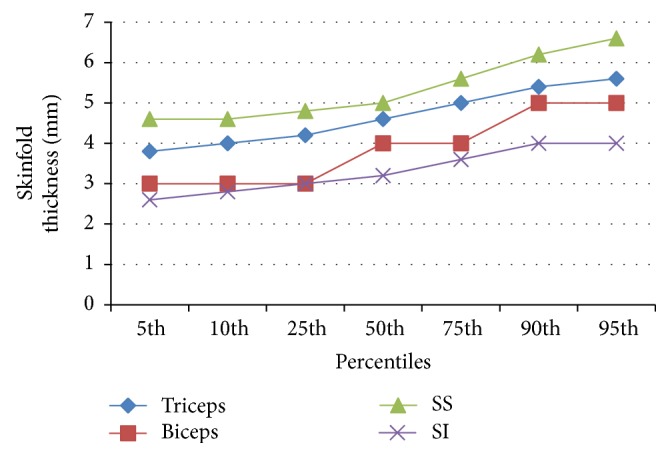
Percentile values for skinfold thickness at various sites in all males.

**Figure 2:**
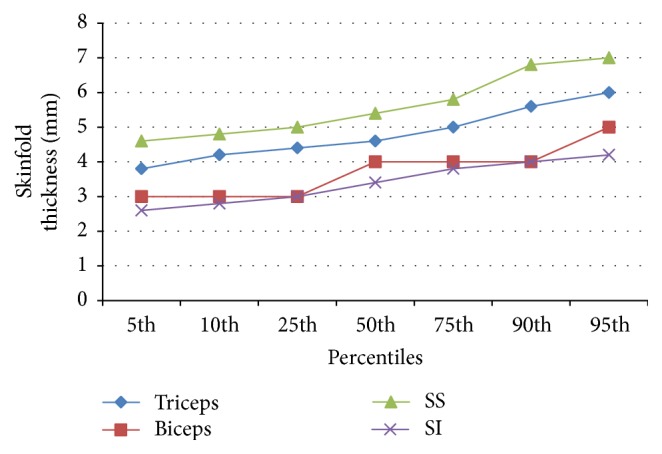
Percentile values for skinfold thickness at various sites in all females.

Figures 1 and 2 show the percentile distribution of skinfold thickness at the triceps, biceps, subscapular, and suprailiac skinfold sites in both sexes. The subscapular skinfold measurement was highest in the neonates followed by the triceps, biceps, and suprailiac sites. The skinfolds in both sexes increased with increasing percentiles.

**Figure 3:**
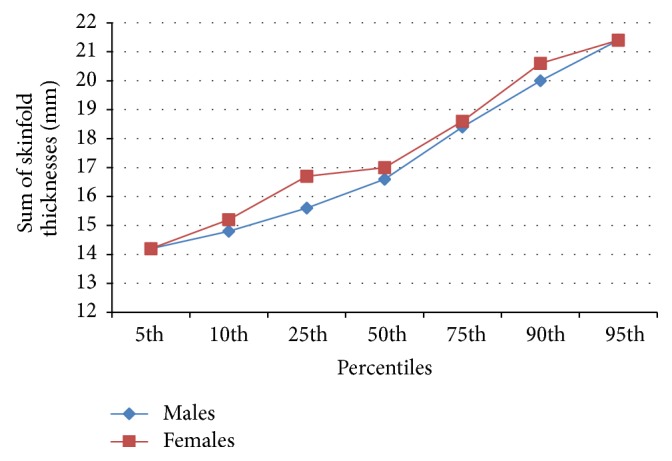
Percentile values for sum of skinfold thicknesses at various sites in males and females.

Selected percentile landmarks were derived for SFT at individual sites for the sum of SFT (Table 5). Across the percentiles, the female neonates had higher skinfolds and the sum of SFT increased with increasing percentile.  Figure 3 shows this graphically.

The correlation matrices for birth weight categories and skinfold measurement are shown in Table 6. The correlation coefficients between birth weight category < 2500 g (LBW) and skinfold measurements ranged from 0.18 to 0.46 while that of birth weight category 2500–4000 g (ABW) and skinfold measurement ranged from 0.46 to 0.66. The correlation coefficient between birth weight category > 4000 g (LABW) and skinfold measurement ranged from 0.27 to 0.37. Correlation was highest in the ABW category. Overall, the highest correlations were between birth weight and subscapular SFT. Correlations were lowest between birth weight and suprailiac SFT.

The mean skinfold thickness values in both sexes according to the birth weight group is shown in Table 7. The mean skinfold thickness increased progressively from the smallest birth weight group to the highest in both sexes. (<2500 g) to the largest (≥4000 g) – *p*-value from ANOVA was consistently <0.001. The mean birth weights of males and females within each birth weight group were similar – generally within 30 g of each other. The females however, had consistently higher mean triceps and subscapular SFT (*p* was at least 0.046). The males had higher values of skinfold thickness at the biceps in those weighing ≥ 3500 g while the females had higher values in babies < 3500 g. However, none of the observed differences reached significant levels (*p* ≥ 0.05). With the exception of low birth weight babies, females had greater mean values of suprailiac SFT than males in all birth weight categories, with significant differences observed in the 3000–3499 g and 3500–3999 g groups. Also, the sum of skinfold thicknesses was consistently higher in females than males being significantly so in the 2500–2999 g, 3000–3499 g, 3500–3999 g groups (*p* was at least 0.001).

The stepwise multiple regression analysis highlighted relationships between birth weight and SFT on adjusting for gender and gestation age. Birth weight was the first variable to enter the model, followed by gender and finally gestational age. Maternal age and other anthropometric indices were excluded by the stepwise procedure. In the final model, regression coefficient was strongest for birth weight and lowest for gestational age. The coefficient of determination (*R* ^2^) showed that the model was able to explain 64% of the variability of the predictors of SFT.

## 4. Discussion

There were gender differences at various points of measurements with females having higher mean figures at the triceps, subscapular, and suprailiac SFT. The sum of subscapular and suprailiac SFT as well as the ratio between central fat and total body fat were also higher in females. A number of studies [23–26] have confirmed these findings. In contrast, however, few studies [27, 28] reported no difference between the two sexes or a higher skinfold measurement in male neonates [29, 30]. It has been suggested that subscapular and suprailiac sites represent central fat while triceps and biceps sites reflect peripheral fat [23]. Thus, females in the current study had higher indices of central fat as has been reported by other workers [23, 24]. The biceps skinfold measurement on the contrary was significantly higher in male neonates. This finding has also been previously documented [31]. The observed gender difference probably reflects hormonal influences. The distribution of body fat is known to be under hormonal control. Thus, differences in hormonal composition may explain the varying patterns of skinfold thickness between males and females [24]. Adipose tissue is now regarded as a complex, highly active metabolic, endocrine organ. It secretes biologically active substances with systemic actions, such as leptin and adiponectin [32] and, besides playing a role in energy homeostasis, it contributes to immune and inflammatory responses. Leptin, which is produced by the placenta and fetus, has been correlated with fat mass and energy balance in neonates and found to vary according to gender [33]. A previous study found that there is a positive correlation between cord blood leptin and gestational age, weight, and ponderal index in newborns, suggesting an association with neonatal growth. Female newborns have higher serum levels of leptin than male ones, indicating that sexual dimorphism in terms of body composition is already present in newborns [34].

The few earlier studies that reported different patterns had relatively smaller sample sizes which may have suppressed manifestations of gender-related patterns.

The mean birth weight of the term neonates in this study is comparable to the range of 3100–3167 g in earlier reports from different centers in the country [35–37] and elsewhere [38]. While it is higher than figures from some other Nigerian reports [39, 40], it is lower than that reported in an earlier study in Lagos [41]. The reason for our figure being lower than that in another Lagos study may be due to the fact that the earlier study was done in a specialist private hospital which caters for mothers of high socioeconomic status. In comparison with studies done elsewhere, our mean birth weight was higher than those reported in Sudan [42] and India [43], respectively, but lower compared to the birth weight of Philadelphia neonates [44]. This difference may be explained by a combination of various factors like race, genetics, maternal nutrition, and socioeconomic status.

Body weight is the best independent predictor of body composition in preterm and term infants, accounting for 84% of the variation in fat mass; sex and length are additional determinants [1]. Our study showed that birth weight accounted for 64% of the variation in fat mass; gender and gestational age were additional determinants. In an Australian study, 36% of the fat mass was accounted for by the birth weight after adjusting for sex and gestation [45]. Body fat increases throughout gestation in both sexes, and female infants have higher body fat percentage than male infants [23]. However, there are limited data about neonatal distribution of subcutaneous body fat. Our finding showed a significant increase with overall fat mass in females and the central to total skinfold was significantly higher beyond 38 weeks of gestation in females compared to males. This is similar to the report by Rodríguez et al. [23].

In comparison with an earlier study done in Ibadan Nigeria [46], the current study recorded higher mean values of triceps, biceps, and subscapular SFT but lower mean suprailiac SFT. It was also observed that babies in the current study were, on the average, 289 g heavier at birth than those involved in the Ibadan study. Considering the strong positive correlation between birth weight and SFT, it is attractive to attribute our higher SFT values to the observed difference in mean birth weight. This argument will however not be sufficient to explain the fact that suprailiac SFT was higher in the Ibadan study despite a recording of lower mean birth weight. It is however instructive to note that the correlation between birth weight and SFT in our study was weaker at the suprailiac site than at other sites. Secondly, the Ibadan study involved only 225 babies in contrast to the current study including over 1000. It is therefore plausible that smaller sample size may to some extent mask expected findings.

Birth weight was significantly related to the triceps, biceps, subscapular, and suprailiac skinfold measurement as well as sum of skinfold measurement. These relationships were strongest with triceps and subscapular sites. This pattern of findings has been previously reported [43, 47] and may explain why these two sites are the commonly used sites for skinfold measurement in children and are the minimum recommended for pediatric research [14, 15]. The females had higher skinfolds even when the neonates were grouped according to birth weight categories, meaning that the increase in weight in the heavier males was due to increased fat-free mass. This implies strongly the need for sex specific references for the neonates.

The percentile charts, developed in the current study, show reference values for skinfold measurements at each site and the sum of skinfold measurement for both sexes. The skinfolds increased with increasing percentile and at every percentile the subscapular skinfolds were the highest followed by the triceps, the biceps, and the suprailiac skinfolds. The female neonates also had higher total sum of skinfold thickness compared to the males at each percentile. These observations have been previously documented in earlier studies [1, 2]. This current study strongly confirms the fact that there are gender differences in neonatal skinfold measurement and hence justifies the development of sex specific percentile charts. However, the development of percentile charts in subsequent research works within the country and region would be necessary in order to define national and regional standards.

In conclusion, the sex specific percentile chart developed for skinfold thickness measurements can be used to detect deviation from the reference population such that infants who are at risk of nutritional or health problems are identified early and get early intervention to reduce morbidity and mortality.

## Figures and Tables

**Table 1:** Birth weight mean ± SD (g), according to gestational age and gender.

Gestational age	Weight (g)		*t* value	*p* value
(Weeks)	Male	Female		
37	3079 ± 0.44	3076 ± 0.43	0.07	0.094
38	3355 ± 0.47	3138 ± 0.48	2.83	0.005^*∗*^
39	3400 ± 0.36	3226 ± 0.41	3.25	0.001^*∗*^
40	3402 ± 0.44	3313 ± 0.44	1.47	0.144
41	3458 ± 0.52	3249 ± 0.45	3.01	0.003^*∗*^

*Overall*	*3339* ± *0.45*	*3200* ± *0.44*	*4.01*	*<0.00*1^*∗*^

^*∗*^Statistically significant *p* < 0.05.

**Table 2:** Skinfold thickness mean ± SD (mm) by gestational age and gender.

Skinfolds	GA	Males	Females	*t* value	*p* value
Triceps	37 wks	4.57 ± 0.56	4.69 ± 0.69	1.65	0.100
	38 wks	4.72 ± 0.85	4.85 ± 0.51	1.35	0.181
	39 wks	4.52 ± 0.40	4.67 ± 0.57	2.32	0.021^*∗*^
	40 wks	4.55 ± 0.51	4.91 ± 0.65	4.44	<0.001^*∗*^
	41 wks	4.53 ± 0.48	4.68 ± 0.50	1.49	0.139
	*Overall*	*4.61 ± 0.58*	*4.74 ± 0.62*	*3.81*	*<0.001* ^*∗*^

Biceps	37 wks	3.79 ± 0.52	3.75 ± 0.58	0.61	0.550
	38 wks	3.95 ± 0.68	3.80 ± 0.38	2.19	0.029^*∗*^
	39 wks	3.88 ± 0.46	3.75 ± 0.45	2.12	0.035^*∗*^
	40 wks	3.81 ± 0.55	3.85 ± 0.46	0.47	0.642
	41 wks	3.85 ± 0.51	3.71 ± 0.50	1.84	0.067
	*Overall*	*3.85 ± 0.56*	*3.77 ± 0.49*	*2.66*	*0.008* ^*∗*^

Subscapular	37 wks	5.16 ± 0.66	5.50 ± 0.89	3.94	<0.001^*∗*^
	38 wks	5.52 ± 0.90	5.65 ± 0.69	1.34	0.181
	39 wks	5.20 ± 0.50	5.47 ± 0.77	3.42	0.001^*∗*^
	40 wks	5.23 ± 0.63	5.62 ± 0.90	3.59	<0.001^*∗*^
	41 wks	5.34 ± 0.75	5.50 ± 0.83	1.42	0.156
	*Overall*	*5.27 ± 0.72*	*5.55 ± 0.81*	*6.23*	*<0.001* ^*∗*^

Suprailiac	37 wks	3.21 ± 0.48	3.37 ± 0.46	2.89	0.004^*∗*^
	38 wks	3.33 ± 0.35	3.48 ± 0.52	2.54	0.012^*∗*^
	39 wks	3.33 ± 0.41	3.61 ± 0.46	4.54	<0.001^*∗*^
	40 wks	3.16 ± 0.39	3.42 ± 0.42	3.66	<0.001^*∗*^
	41 wks	3.18 ± 0.40	3.56 ± 0.57	5.28	<0.001^*∗*^
	*Overall*	*3.32 ± 0.47*	*3.41 ± 0.48*	*3.29*	*<0.001* ^*∗*^

^*∗*^Significant *p* < 0.05.

**Table 3:** Distribution of central skinfolds according to sex and gestational age.

Gestational age (weeks)	Central skin folds (SBS + SPS)			
	Male Mean ± SD	Female Mean ± SD	*t* value	*p* value
37	8.53 ± 1.01	8.71 ± 1.21	1.45	0.148
38	9.01 ± 1.33	9.02 ± 0.94	0.05	0.958
39	8.49 ± 0.81	9.08 ± 1.14	4.32	<0.001^*∗*^
40	8.39 ± 0.95	9.06 ± 1.20	4.39	<0.001^*∗*^
41	8.52 ± 1.07	9.06 ± 1.29	3.20	0.002^*∗*^

*Overall*	*8.59 (1.10)*	*8.97 (1.16)*	*5.77*	*<0.001* ^*∗*^

^*∗*^Significant *p* < 0.05; SBS = subscapular skinfold; SPS = suprailiac skinfold.

**Table 4:** The central to total skinfold ratio according to gender and gestational age.

Gestational age (weeks)	Central to total skinfold ratio			
	Male Mean ± SD	Female Mean ± SD	*t* value	*p* value
37	0.5050 ± 0.02	0.5082 ± 0.07	1.37	0.175
38	0.5097 ± 0.01	0.5142 ± 0.06	0.83	0.408
39	0.5036 ± 0.01	0.5075 ± 0.11	2.93	0.004^*∗*^
40	0.5010 ± 0.01	0.5341 ± 0.02	3.22	0.001^*∗*^
41	0.4989 ± 0.01	0.5285 ± 0.06	4.59	<0.001^*∗*^

*Overall*	*0.5049 (0.03)*	*0.5166 (0.56)*	*4.46*	*<0.001* ^*∗*^

^*∗*^Significant *p* < 0.05.

**Table 5:** Percentile distribution for the sum of skinfold measurements in the neonates.

	Sex	5th	10th	25th	50th	75th	90th	95th
*Sum of SFT*								
37 wks	M	13.4	14.2	15.2	16.0	18.0	19.2	20.1
	F	13.8	14.4	15.8	16.8	18.2	20.9	22.0
38 wks	M	13.6	14.5	15.6	17.0	18.3	20.8	21.3
	F	15.2	15.8	16.6	17.4	18.8	21.8	22.6
39 wks	M	14.4	15.2	15.8	16.6	18.6	19.2	20.0
	F	15.0	15.4	16.0	16.9	19.2	21.2	21.7
40 wks	M	14.2	14.5	15.4	16.2	18.4	19.4	19.7
	F	14.8	15.6	16.3	17.1	19.0	20.2	20.8
41 wks	M	14.0	14.2	15.6	16.4	18.0	20.4	20.6
	F	14.6	14.8	16.0	17.2	18.6	20.7	21.6

*Overall*		*14.2*	*14.8*	*15.8*	*16.8*	*18.6*	*20.2*	*21.2*

**Table 6:** Correlation coefficients between birth weight categories and skinfold measurements at various sites.

Birth weight	Triceps	Biceps	S/scapular	Suprailiac
	SFT	SFT	SFT	SFT
<2500 g (LBW)	0.40^*∗∗*^	0.32^*∗∗*^	0.46^*∗∗*^	0.18^*∗∗*^
2500–4000 g (ABW)	0.65^*∗∗*^	0.63^*∗∗*^	0.66^*∗∗*^	0.46^*∗∗*^
>4000 g (LABW)	0.32^*∗∗*^	0.31^*∗∗*^	0.37^*∗∗*^	0.27^*∗∗*^
Overall	0.77^*∗∗*^	0.71^*∗∗*^	0.76^*∗∗*^	0.55^*∗∗*^

^*∗∗*^ *p* < 0.05, LBW: low birth weight, ABW: appropriate birth weight, and LABW: large birth weight.

**Table 7:** Mean skinfold thickness values ± SD in males and females according to birth weight groups.

Range	Birth weight g	Triceps	Biceps	Subscapular	Suprailiac	Sum	*n*
	M ± SD	M ± SD	M ± SD	M ± SD	M ± SD	M ± SD	
*≤2500*							
Males	2.31 ± 0.14	3.52 ± 0.41	2.88 ± 0.60	4.20 ± 0.62	3.04 ± 0.38	13.60 ± 1.4	22
Female	2.29 ± 0.15	3.80 ± 0.36	2.95 ± 0.40	4.55 ± 0.27	2.62 ± 0.30	13.88 ± 1.0	23
*t*	0.13	2.17	0.41	2.15	3.65	0.66	
*p*	0.90	*0.04*	0.69	*0.04*	*0.001*	0.52	
*2500–2999*							
Male	2.68 ± 0.14	4.10 ± 0.37	3.42 ± 0.50	4.77 ± 0.32	3.05 ± 0.36	15.31 ± 1.2	90
Female	2.69 ± 0.15	4.29 ± 0.35	3.47 ± 0.53	4.97 ± 0.35	3.11 ± 0.37	15.82 ± 1.1	124
*t*	0.16	3.82	0.71	4.36	1.20	3.23	
*p*	0.87	*<0.001*	0.48	*<0.001*	0.23	*0.001*	
*3000–3499*							
Male	3.21 ± 0.14	4.43 ± 0.30	3.70 ± 0.49	4.95 ± 0.35	3.18 ± 0.40	16.25 ± 1.1	258
Female	3.19 ± 0.15	4.60 ± 0.31	3.75 ± 0.46	5.31 ± 0.37	3.37 ± 0.39	16.91 ± 1.3	255
*t*	0.49	2.00	1.19	11.34	5.46	6.11	
*p*	0.62	*0.046*	0.23	*<0.001*	*<0.001*	*<0.001*	
*3500–3999*							
Male	3.64 ± 0.13	4.96 ± 0.43	4.25 ± 0.56	5.75 ± 0.54	3.53 ± 0.43	18.40 ± 1.74	185
Female	3.61 ± 0.13	5.28 ± 0.51	4.14 ± 0.43	6.29 ± 0.68	3.76 ± 0.36	19.48 ± 1.49	129
*t*	2.01	5.83	1.97	7.85	5.14	5.90	
*p*	*0.045*	*<0.001*	0.05	*<0.001*	*<0.001*	*<0.001*	
*≥4000*							
Male	4.26 ± 0.25	5.53 ± 0.47	4.65 ± 0.52	6.59 ± 0.59	3.87 ± 0.33	20.55 ± 1.30	49
Female	4.15 ± 0.28	5.82 ± 0.65	4.43 ± 0.55	7.15 ± 0.82	3.92 ± 0.46	21.01 ± 2.84	34
*t*	1.89	2.30	1.88	3.52	0.56	0.92	
*p*	0.06	*0.02*	0.06	*<0.001*	0.58	0.36	

ANOVA							
*Males*							
*F*-statistic	1351.6	209.4	91.8	243.3	56.9	213.2	
*p* value	<0.001	<0.001	<0.001	<0.001	<0.001	<0.001	
*r*		*0.994*	*0.990*	*0.982*	*0.958*	*0.992*	
*p*		*0.001*	*0.001*	*0.001*	*0.010*	*0.001*	
*Females*							
*F*-statistic	1004.2	192.7	107.5	227.0	80.3	175.8	
*p* value		<0.001	<0.001	<0.001	<0.001	<0.001	
*r*		*0.800*	*0.696*	*0.813*	*0.625*	*0.782*	
*p* *value*		*<0.001*	*<0.001*	*<0.001*	*<0.001*	*<0.001*	

**Table 8:** Stepwise regression of predictors of skinfold thickness.

Models	Predictors	Standardized coefficients	*t*	Sig.	*R*	*R* ^2^
(1)	Birth wt (KG)	0.760	39.893	0.000	0.760	0.577

(2)	Birth wt (KG)	0.780	42.250	0.000	0.780	0.608
	Gender	0.177	9.574	0.000		

(3)	Birth wt (KG)	0.819	45.059	0.000	0.799	0.638
	Gender	0.190	10.650	0.000		
	Gestational age	−0.178	−9.870	0.000		

Wt = weight measured in kilogram.
